# Prediction of plant secondary metabolic pathways using deep transfer learning

**DOI:** 10.1186/s12859-023-05485-9

**Published:** 2023-09-19

**Authors:** Han Bao, Jinhui Zhao, Xinjie Zhao, Chunxia Zhao, Xin Lu, Guowang Xu

**Affiliations:** 1 CAS Key Laboratory of Separation Science for Analytical Chemistry, Dalian Institute of Chemical Physics, Chinese Academy of Sciences, Dalian, 116023 People’s Republic of China; 2 University of Chinese Academy of Sciences, Beijing, 100049 People’s Republic of China; 3 Liaoning Province Key Laboratory of Metabolomics, Dalian, 116023 People’s Republic of China

**Keywords:** Metabolic pathway prediction, Plant secondary metabolism, Deep learning, Transfer learning, Graph Transformer

## Abstract

**Background:**

Plant secondary metabolites are highly valued for their applications in pharmaceuticals, nutrition, flavors, and aesthetics. It is of great importance to elucidate plant secondary metabolic pathways due to their crucial roles in biological processes during plant growth and development. However, understanding plant biosynthesis and degradation pathways remains a challenge due to the lack of sufficient information in current databases. To address this issue, we proposed a transfer learning approach using a pre-trained hybrid deep learning architecture that combines Graph Transformer and convolutional neural network (GTC) to predict plant metabolic pathways.

**Results:**

GTC provides comprehensive molecular representation by extracting both structural features from the molecular graph and textual information from the SMILES string. GTC is pre-trained on the KEGG datasets to acquire general features, followed by fine-tuning on plant-derived datasets. Four metrics were chosen for model performance evaluation. The results show that GTC outperforms six other models, including three previously reported machine learning models, on the KEGG dataset. GTC yields an accuracy of 96.75%, precision of 85.14%, recall of 83.03%, and F1_score of 84.06%. Furthermore, an ablation study confirms the indispensability of all the components of the hybrid GTC model. Transfer learning is then employed to leverage the shared knowledge acquired from the KEGG metabolic pathways. As a result, the transferred GTC exhibits outstanding accuracy in predicting plant secondary metabolic pathways with an average accuracy of 98.30% in fivefold cross-validation and 97.82% on the final test. In addition, GTC is employed to classify natural products. It achieves a perfect accuracy score of 100.00% for alkaloids, while the lowest accuracy score of 98.42% for shikimates and phenylpropanoids.

**Conclusions:**

The proposed GTC effectively captures molecular features, and achieves high performance in classifying KEGG metabolic pathways and predicting plant secondary metabolic pathways via transfer learning. Furthermore, GTC demonstrates its generalization ability by accurately classifying natural products. A user-friendly executable program has been developed, which only requires the input of the SMILES string of the query compound in a graphical interface.

**Supplementary Information:**

The online version contains supplementary material available at 10.1186/s12859-023-05485-9.

## Background

Plant secondary metabolites are metabolic intermediates and products, which are considered to be non-essential for the growth and survival of the organism. However, they not only participate in environmental responses like stress resistance [1, 2] and disease resistance [3, 4], but also are active ingredients of many herbal medicines [5]. Research on plant secondary metabolism can guide the cultivation of excellent crops with desirable traits and contribute to the discovery of novel drugs for human diseases.

Metabolomics is a powerful tool for exploring the production and functions of secondary metabolites [6]. Through differential metabolite analysis, changes in metabolic pathways provide valuable insights into underlying biological mechanisms, such as pathogenic disease [7] and crop selection [8]. Pathway enrichment analysis based on metabolomics data may be biased. It is primarily due to the limited current knowledge of plant secondary metabolic pathways [9], and certain pathways have not been included in existing databases due to the outdatedness and maintenance requirements [10]. Exploring these pathways through biological experiments is time-consuming and resource-intensive. Therefore, it is crucial to develop new ways of improving the understanding of plant secondary metabolism.

Several machine learning algorithms have been reported to predict the metabolic pathways of a compound, including traditional machine learning algorithms and deep learning models. In early research, traditional algorithms such as the Nearest Neighbor Algorithm [11] and Adaboost [12] were adopted to classify the compounds into a single pathway category, ignoring the possibility that a compound might belong to multiple metabolic pathways. Later, multi-target models were proposed based on chemical interaction [13, 14] to predict the order of predicted metabolic pathway classes of the query compound. A bioinformatics tool, TrackSM [15], was developed to extract scaffolds from compounds in the same metabolic pathways. It assumed that scaffolds (substructures) are associated with corresponding metabolic pathways. Besides, the Support Vector Machine (SVM), a powerful algorithm, has been widely used in many fields, including protein structural class prediction [16]. In the context of metabolic pathway prediction, several studies have tackled the challenge of the multi-label classification by converting it into a binary classification task [17–20].

Recently, deep learning models have been applied to predict metabolic pathways. The first deep learning model adopted in predicting metabolic pathways was a Graph Convolutional Network (GCN) [21]. This prediction engine only requires the simplified molecular-input line-entry system (SMILES) [22] string of the query compound and outperformed traditional machine learning algorithms such as Random Forest (RF) model. Afterward, the Graph Attentional Network (GAT) has been reported to have better performance [23, 24]. However, most prediction results are limited to the 11 pathway classes in the Kyoto Encyclopedia of Genes and Genomes (KEGG) [25], which is not refined enough. Although some investigation has been reported to classify compounds into sub-classes of metabolic pathways [26], plentiful negative samples were introduced. Additionally, there is no graphical user interface available for users.

Machine learning and deep learning models have demonstrated their potential in predicting metabolic pathways, which can greatly improve our understanding of plant secondary metabolism. However, these models require further refinement and expansion of their applications. Ongoing developments in Graph Neural Networks (GNN) and Convolution Neural Networks (CNN) are expected to contribute to this progress. Moreover, transfer learning can effectively improve the efficiency of model construction and solve the problem of insufficient data. In this paper, we propose a transfer learning approach using a pre-trained hybrid deep learning architecture that combines graph transformer [27] and CNN (GTC) to predict plant metabolic pathways. Overall, the primary contributions of this article are as follows:

*A deep ensemble learning model* The architecture of our proposed model, GTC, comprises two distinct blocks. A GNN-based block captures the structural features of the molecule by treating it as a graph, and a CNN-based block learns from the text information of the SMILES string. By combining these two representations, GTC provides a more comprehensive representation of molecules, leading to outperforming state-of-the-art methods in predicting metabolic pathways.

*Transfer strategy of fine-tuning* To solve the problem of insufficient data on plant secondary metabolic pathways, transfer learning is implemented. After obtaining high performance of the pre-training model on the KEGG dataset, GTC is fine-tuned to perform the accurate prediction for plant secondary metabolic pathways.

*Model generalization* The modular architecture of GTC allows for easy adaptation to various molecular prediction tasks beyond metabolic pathway classification. The model can be adapted to predict other molecular properties, such as classifying natural products, by simply modifying the output layer accordingly.

## Methods

### Datasets

To implement a transfer learning process, separate datasets are required for both the target task and the source task, respectively. In this study, four datasets were used (Additional file 2: Table S1). Both Dataset A (Additional file 2: Table S2) and Dataset B (Additional file 2: Table S3) were created for the target task, while Dataset C (Additional file 2: Table S4) served as a benchmark dataset for the source task. Besides, Dataset D (Additional file 2: Table S5) was generated to evaluate the generalization ability of the GTC model. The detailed description is as follows:

Dataset A houses 2028 plant-derived compounds, represented by SMILES strings and labeled with 18 pathway labels. The information was collected from Plant Metabolic Network (PWN) [28] in October 2022. The 18 sub-classes are labeled as ‘0’, ‘1’, …, ‘17’ and encompass secondary metabolite biosynthesis and degradation pathways, including aroma compound biosynthesis, fatty acid derivative biosynthesis, … and terpenoid degradation (Additional file 2: Table S6). Since a single compound may belong to several metabolic pathways (Additional file 1: Fig. S1 and Fig. S2), it is a multi-label task. Besides, the unbalanced distribution of the 2028 compounds is observed in Additional file 2: Table S6. In the presence of an unbalanced distribution, deep learning model from scratch may overly focus on learning the majority classes, leading to increased errors when predicting the minority classes. To address the challenge, a transfer learning approach was adopted to leverage knowledge from a related dataset through pre-training. Our model benefits from pre-trained representations, ultimately resulting in improved performance on minority classes.

Dataset B consists of 577 plant-derived compounds, which are classified into the same 18 pathway categories as Dataset A. The dataset was extracted from the updated PWN database in February 2023 and retained only the newly added compounds absent in Dataset A. It is noted that Dataset B is excluded from the training process and only served for the final testing evaluation. Dataset C comprises 5288 compounds and 11 pathway classes in the KEGG database, downloaded in December 2022. Similarly, the compounds are also represented by SMILES strings. The 11 classes of metabolic pathways include carbohydrate metabolism, energy metabolism, lipid metabolism, and so on. (Additional file 2: Table S7). Dataset C has great similarity in task and domain with Dataset A, as both datasets involve chemical structures as input and labels related to metabolic pathways. Additionally, Dataset C has been carefully annotated by domain experts, which ensures data reliability. Additional file 1: Fig. S3 and Additional file 2: Table S7 show that Dataset C provides enough data instances. Therefore, it is reasonable to consider Dataset C as the source data for transfer learning. Besides, such a dataset from the KEGG database was widely used in previous studies for the prediction of metabolic pathways. Dataset D contains 78,305 natural products, which are labeled by 7 Pathways. It was originally collected by the researchers of NPClassifier [29], which was reported for the classification of natural products by using counted Morgan fingerprints as input. The primary data with 78,336 compounds were downloaded at https://github.com/mwang87/NP-Classifier in March 2023. The deduplication was conducted and created Dataset D, which was used to test the generalization ability of the GTC model.

### Model construction

**Fig. 1:**
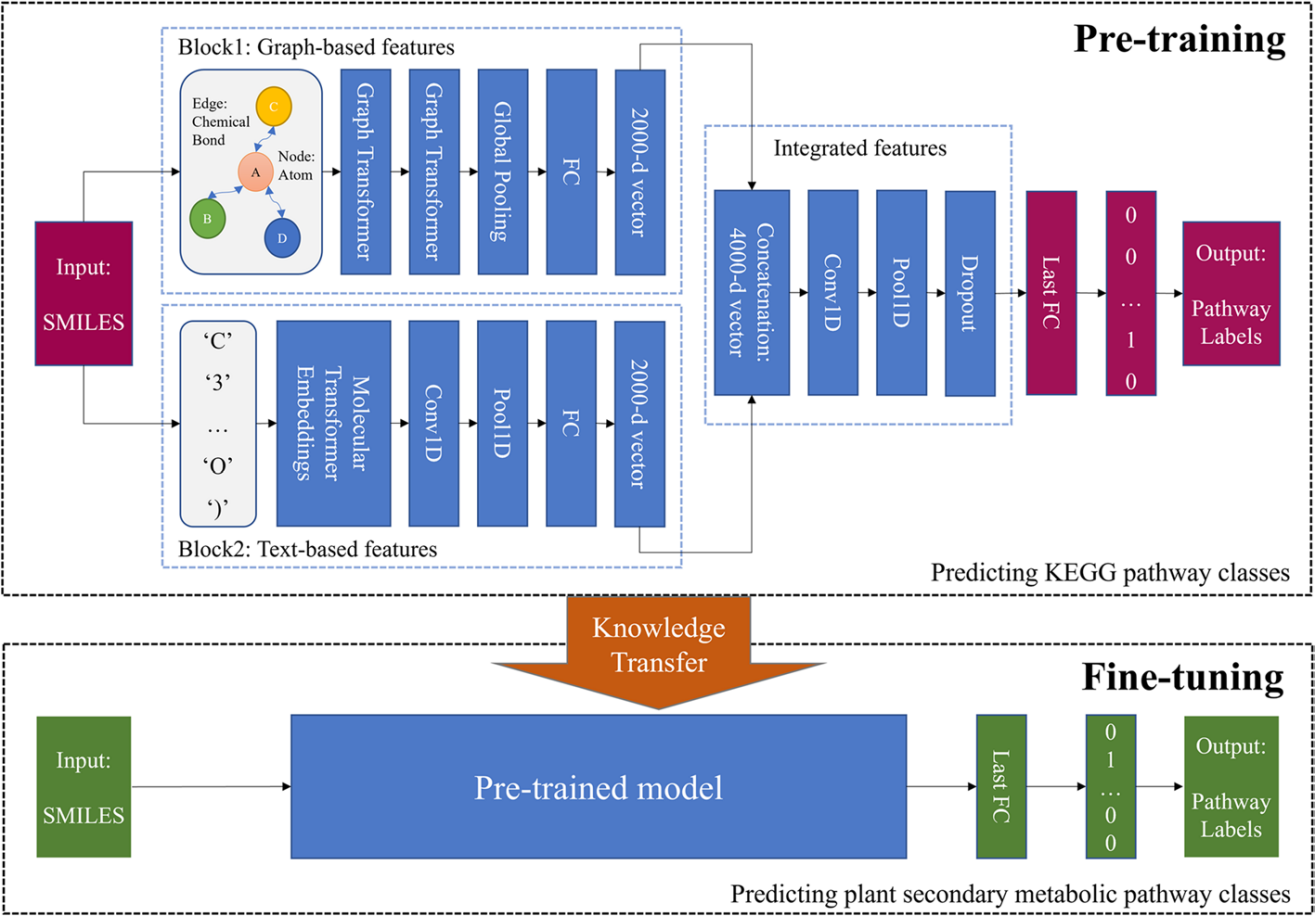
Flowchart of the proposed GTC. The whole pipeline contains two processes: pre-training and fine-tuning. The model is initially constructed on the KEGG dataset and then leverages the learned knowledge to predict plant secondary metabolic pathways. Conv1D stands for one-dimensional convolutional layer; Pool1D stands for one-dimensional max-pooling layer; FC stands for fully connected layer

The deep learning model, GTC, is an ensemble of a graph transformer network and a CNN. Figure 1 shows the overall framework of GTC as well as the pipeline of the whole transfer learning process, which is further explained in the section **Transfer learning**. The model comprises two separate blocks that capture molecular features from different perspectives. The first block focuses on extracting graph-based molecular structural features, while the second block learns text-based molecular structural information. By combining these two representations, the artificial neural network can comprehensively and effectively learn molecular structures. The integrated features are then passed through an output layer for prediction.

*Block1* In this block, a molecular is seen as a graph composed of nodes and edges. Here, the atom is the node, while the chemical bond is the edge. Each atom *i* is thought to have connections with its neighbor *j*. Specifically, it is given that node features **X** = { **x**_*1*_, **x**_*2*_, **x**_*3*_, …, **x**_*n*_}. Partially drawing on existing research [24, 30], the node feature **x**_*i*_ is initialized with the combination of the information of the atom symbol, the number of adjacent atoms, the number of adjacent hydrogens, the formal charge of the atom, the implicit valence of the atom, the chiral type of the atom, whether the atom is in a ring and whether the atom is in an aromatic structure, using RDKIT [31]. In this way, the *i*th node feature **x**_*i*_ and the *j*th node feature **x**_*j*_ are obtained. Corresponding to the self-attention mechanism in the well-known Transformer[32], there are three important elements in graph transformer: *Query*, *Key*, and *Value*, which are calculated by:

$$Query={\mathbf{W}}_{c,q}{\mathbf{x}}_{i} ,$$

$$Key={\mathbf{W}}_{c,k}{\mathbf{x}}_{j} ,$$

$$Value={\mathbf{W}}_{c,v}{\mathbf{x}}_{j} ,$$

where **W**_*c,q*_, **W**_*c,k*_, and **W**_*c,v*_ are three weighing matrices. Basically, in a Graph Transformer layer, the node feature is updated by aggregating the features of its neighboring atoms. With multi-head attention adopted, for the *c*th attention head, **x**_*i*_ is updated by the following propagation rule:

$${\mathbf{x}}_{c, i}^{\prime}={\mathbf{W}}_{c,l}{\mathbf{x}}_{i}+{\sum }_{j\in N(i)}\left({\alpha }_{c,i,j}{\mathbf{W}}_{c,v}{\mathbf{x}}_{j}\right) ,$$

where **W**_*c,l*_ is another weighing matrix and *α*_*c,i,j*_ is the attention coefficient, which is computed by:

$${\alpha }_{c,i,j}={\mathrm{Softmax}\left(\frac{{\left({\mathbf{W}}_{c,q}{\mathbf{x}}_{i}\right)}^{\mathrm{T}}({\mathbf{W}}_{c,k}{\mathbf{x}}_{j})}{\sqrt{d}}\right)},$$

where *d* is the dimension of **x**_*i*_. After being updated by each attention head, the node feature is concatenated by:

$${\mathbf{x}}_{i}^{\prime}={\parallel }_{c=1}^{C}{(\mathbf{x}}_{c,i}{\prime}) ,$$

where *C* is the total number of attention heads and ∥ is the operator of concatenating the vectors. Subsequently, such two methods as global mean pooling and global max pooling are adopted in parallel in the Global Pooling layer. Finally, through a fully-connected layer, a molecule is represented by a 2000-dimensional vector.

*Block2* In this block, the SMILES string of a molecule is input in text form. The neural network of MolecularTransformerEmbeddings [33] is used to obtain the molecular representation vector, in which every character has its specific attention weights. Therefore, this vector encodes the structural characteristics of the molecule. Subsequently, the vector passes through a one-dimensional convolution layer and a one-dimensional pooling layer. Finally, similar to Block1, a molecule is represented by a 2000-dimensional vector through a fully-connected layer.

Extracted from the two blocks, the features of a molecule are concatenated as a 4000-dimensional vector. The information is learned based on a CNN akin to Block2 and a dropout layer is employed to prevent the model over-fitting. As the output layer, the last fully connected layer (Last FC) provides a set of probabilities that the compound belongs to each metabolic pathway. The final prediction is made by utilizing a threshold value of 0.5. As for a bit-string of ‘0001…1000’ given, ‘1’ at the *p*th position reflects that the metabolite belongs to the *p*th metabolic pathway, while ‘0’ suggests that it does not belong to that pathway.

To get a pre-trained model for subsequent transfer learning, several variant models were built for performance comparison. Different ways of message passing between nodes in graphs affect the performance of GNN. Therefore, three variants were created by replacing the GNN layer in Block1 while keeping the small convolution kernel in Block2 unchanged including GCN combining with CNN (GCN + CNN), GAT combining with CNN (GAT + CNN), SuperGAT [34] combining with CNN (SuperGAT + CNN). It should be noted that common GCN and GAT have been previously utilized in the prediction of metabolic pathways, whereas SuperGAT has not been applied to this task before. Additionally, a fivefold cross-validation was conducted. The model performance is evaluated by accuracy, precision, recall, and F1_score. Here, we choose micro F1_score. Additional file 1 provides an exhaustive description and discussion of these evaluation metrics, including implementation specifics.

### Transfer learning

**Fig. 2:**
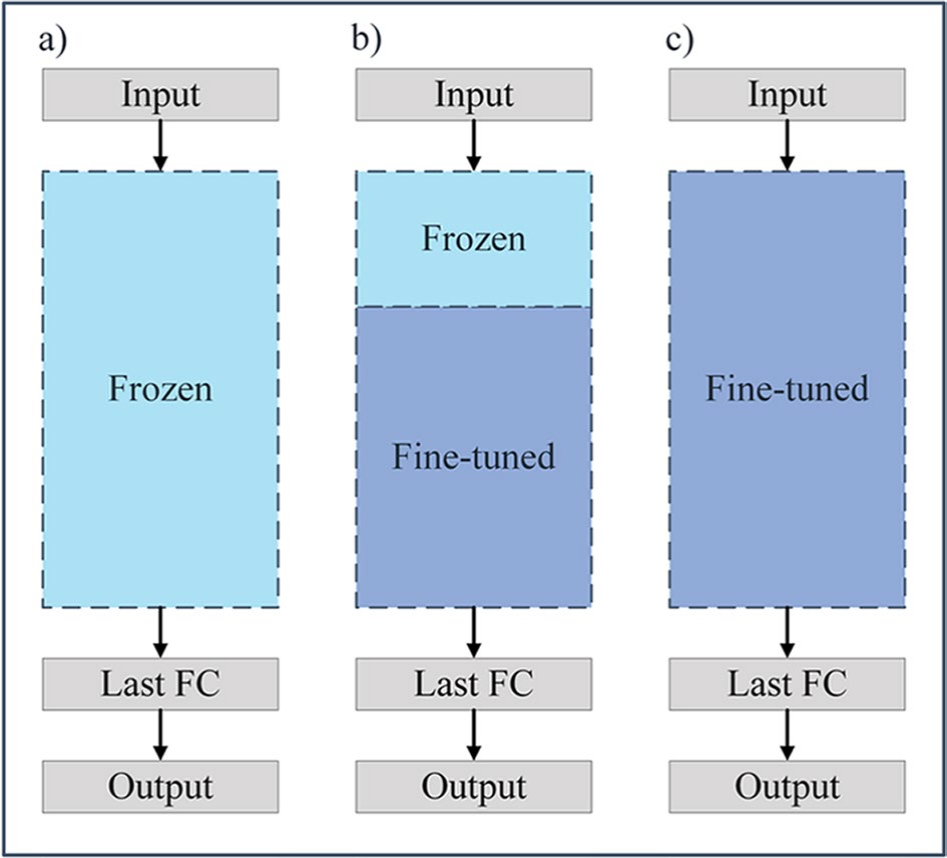
Three common strategies utilized in transfer learning. **a** All the pre-trained weights are frozen and only the last fully connected layer (output layer) is changed. **b** Some layers are fine-tuned and the others are frozen. **c** The entire model is fine-tuned with the pre-trained weights

GTC undergoes an initial pre-training process on Dataset C to acquire knowledge, which is subsequently transferred to predict plant secondary metabolic pathways (Fig. 1). During the fivefold cross-validation, the model with the highest accuracy is chosen. As described in Fig. 2, there are three common strategies for transfer learning. In the first pattern, all the pre-trained weights are frozen and only the last fully connected layer (output layer) is changed. The other two patterns use fine-tuning, which involves updating a deployed learning model with a smaller learning rate. In the second one, some layers are fine-tuned and the others are frozen. In the third strategy, the entire model is fine-tuned with the pre-trained weights. Based on these three patterns, five concrete transfer strategies have been implemented as follows:

- *Entire model frozen*: the pre-trained weights were frozen in the entire model.
- *Block1 frozen*: the pre-trained weights were frozen in Block1.
- *Block2 frozen*: the pre-trained weights were frozen in Block2.
- *Block1* + *Block2 frozen*: the pre-trained weights were frozen in both Block1 and Block2.
- *No module frozen*: fine-tune the entire model with the pre-trained weights.

The learning rate decreases from ‘0.0003’ (default) to ‘0.0001’ in the five transfer strategies. In addition, as for the three methods of Block1 frozen, Block2 frozen and Block1 + Block2 frozen, the pre-trained weights in the fully connected layer are not frozen.

## Results and discussion

### Performance comparison of several machine learning models on the benchmark dataset

Four deep learning models are constructed, including GTC and its three variant models: GCN + CNN, GAT + CNN, and SuperGAT + CNN. Their performances are compared with previously reported methods. Among the related research, only two studies have made their codes publicly available. One study [21] proposed RF-based and GCN-based models for metabolic pathway prediction. The other study developed MLGL-MP [24], which utilized both GAT and GCN for the compound encoder and GCN for the pathway encoder. We use their source codes to retrain the three aforementioned models. Five-fold cross-validation experiments are conducted for all seven models.

As seen in Table 1, GTC outperforms the other models, yielding an accuracy of 96.75%, precision of 85.14%, recall of 83.03%, and F1_score of 84.06%. A two-tailed student *t*-test is performed to compare GTC with each of the other six models. The results show that the four scores of GTC are statistically significantly better than the three previously published methods, except for precision with MLGL-MP. As for our models, significant differences exist between GTC and GCN + CNN. However, no significant differences are observed between GTC and GAT + CNN or between GTC and SuperGAT + CNN, respectively. It should be noted that both GAT + CNN and SuperGAT + CNN use the attention mechanism in their graph neural network. It implies that the attention mechanism plays a crucial role in feature extraction and aggregation in the graph. The attention coefficient in graph transformer is calculated more effectively, particularly for our task.

**Table 1:** Performance of GTC and other machine learning models

Methods	Accuracy (%)	Precision (%)	Recall (%)	F1_score (%)
RF-based	95.66 ± 0.16***	70.95 ± 0.89***	69.38 ± 0.84***	70.16 ± 0.86***
GCN-based	95.94 ± 0.14***	80.81 ± 0.36***	79.47 ± 1.20**	80.14 ± 0.71***
MLGL-MP	96.45 ± 0.15*	84.67 ± 1.36	80.10 ± 0.78**	82.32 ± 0.60*
GCN + CNN	96.05 ± 0.14***	82.26 ± 0.66**	78.67 ± 1.53**	80.41 ± 0.75***
GAT + CNN	96.51 ± 0.27	83.99 ± 1.73	81.74 ± 0.73	82.84 ± 1.10
SuperGAT + CNN	96.63 ± 0.24	84.30 ± 1.46	82.75 ± 0.81	83.52 ± 1.06
GTC	**96.75 ± 0.21**	**85.14 ± 1.42**	**83.03 ± 0.92**	**84.06 ± 0.85**

The values represent the mean ± standard deviation obtained through fivefold cross-validation. The best value on the metric is highlighted in bold; The symbols *, **, and *** mean that the performance of GTC is significantly better in the* t*-test at the p-values less than 0.05, 0.01, and 0.001, respectively.

### Ablation studies

For an ensemble learning model, ablation studies are significant to study the contribution of each component to the whole system. To evaluate the effectiveness of the components of GTC, ablation studies were conducted on three variants of the model proposed as follows. The first variant removed CNN layers after concatenation of the vectors learned by Block1 and Block2. The second and the third were in lack of Block2 and Block1, respectively.

**Fig. 3:**
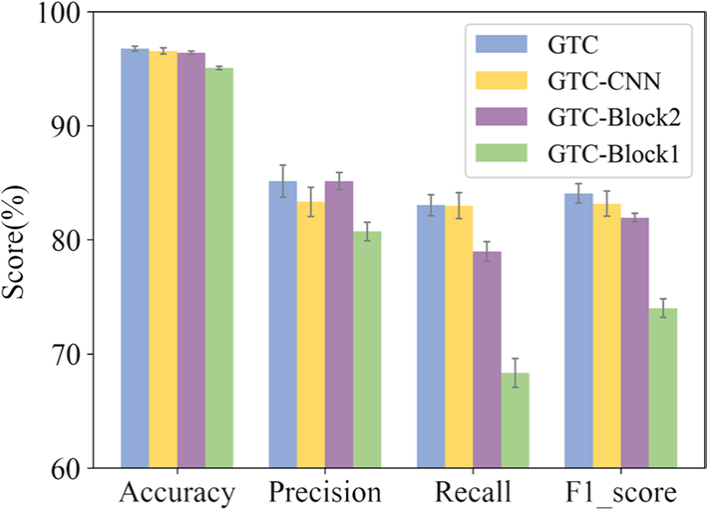
The effects of Block1, Block2, and CNN layers on performance efficacy after the concatenation of vectors learned by Block1 and Block2, respectively

As seen in Fig. 3, the intact GTC model achieves the best performance over all four evaluation metrics. Each of the three components contributed differently to the learning model. The removal of CNN layers after the concatenation of the two vectors doesn’t have a significant impact. Since features are extracted through Block1 and Block2, a fully connected layer could complete a decent output result. A similar performance is produced for the removal of Block2. However, removing Block1 results in a drastic underperformance. Such results indicate that the graph transformer network plays a crucial role in our model. In general, all components contribute to the prediction task in varying degrees, so none of them should be eliminated.

### Performance of transfer learning

To evaluate the effectiveness of transfer learning, we perform the fivefold cross-validation experiments on Dataset A, using the five strategies described in the section **Transfer learning**, **Methods**. It can be seen that freezing the pre-trained weights in Block2 (referred to as “Block2 frozen”) yields an accuracy of 98.30%, precision of 88.44%, recall of 84.36%, and F1_score of 86.34% (Table 2). All four metrics of accuracy, precision, recall, and F1_score achieve the best, suggesting that “Block2 frozen” represents the most effective model for our task. Among the five transfer training strategies, fine-tuning the entire model and fine-tuning the model with Block2 frozen performed extremely close in terms of the four evaluation metrics. The input of Block2 is the embedding results through the neural network named MolecularTransformerEmbeddings [33]. After pre-training on Dataset C, Block2 was adjusted to the task of multi-label prediction. Retraining Block2 is unnecessary due to the increase of additional computational costs and time consumption without substantial gains in performance. A similar conclusion is also drawn from comparing the freezing of Block1 and the freezing of both Block1 and Block2. However, freezing Block1 adversely affects model performance, which is consistent with the results of the ablation study. Due to the core role of Block1, fine-tuning Block1 is recommended to adapt it to the new prediction task. It should also be noted that freezing the entire model degraded model performance abruptly, possibly because the chemical structures and label distribution differ drastically between Dataset A and Dataset B. Therefore, the strategy of fine-tuning is a better option. In summary, it is an appropriate strategy to fine-tune the transfer model with the pre-trained weights in Block2 frozen, in terms of predicting plant secondary metabolic pathways.

**Table 2:** Performance of different transfer strategies for plant secondary metabolic pathway prediction

Strategy	Accuracy (%)	Precision (%)	Recall (%)	F1_score (%)
Entire model frozen	97.04 ± 0.18	80.23 ± 0.99	70.90 ± 2.17	75.26 ± 1.44
Block1 frozen	98.16 ± 0.20	86.99 ± 2.29	83.49 ± 1.10	85.20 ± 1.53
Block2 frozen	**98.30 ± 0.15**	**88.44 ± 1.59**	**84.36 ± 1.17**	**86.34 ± 1.07**
Block1 + Block2 frozen	98.15 ± 0.19	86.99 ± 2.26	83.45 ± 0.94	85.17 ± 1.40
No module frozen	98.29 ± 0.15	88.23 ± 1.55	84.32 ± 1.31	86.22 ± 1.11

The values represent the mean ± standard deviation obtained through fivefold cross-validation. The best value on the metric is highlighted in bold

Then, the same strategy is implemented to conduct the final test on Dataset B, which is not involved in model training. All the 2028 molecules in Dataset A are used as the training set. After 300 epochs, the prediction model is saved, through which the prediction results are obtained. Moreover, we investigate the influences of data augmentation, an alternative method often used to address data insufficiency and imbalance. Here, it is hypothesized that the two molecules with high structural similarity would be involved in the same metabolic pathway. Data augmentation methods α and β involve the replacement of methoxy groups with hydroxy groups and the replacement of hydroxy groups with methoxy groups [29]. α is augmentation once and β is applied three times. Data augmentation methods γ and θ leverage molecular fingerprint similarity, calculating the Tanimoto coefficient between the 2028 molecules in Dataset A and the molecules in the COCONUT database [35]. γ and θ choose fingerprint similarity between 0.99–1 and 0.96–1, respectively. The value of 0.96 represents the average Tanimoto coefficient in the datasets created by α and β. As described in Table 3, the transfer learning approach exhibits decent performance. The accuracy is 97.82%, while precision, recall, and F1_score are all at 83.74%. In contrast, the impact of four data augmentation is negligible. However, it is noteworthy that the recall value of 84.32% indicates a decrease in the number of false negatives when the number of training data pieces reached 52,530. Thus, considering both model performance and computational cost, transfer learning outperformed data augmentation in predicting plant secondary metabolic pathways.

**Table 3:** Performance comparison between transfer learning and several data augmentation methods for plant secondary metabolic pathway prediction

Methods	Compounds	Accuracy (%)	Precision (%)	Recall (%)	F1_score (%)
Transfer Learning	2028	**97.82**	**83.74**	83.74	**83.74**
Data augmentation α	7666	97.80	83.10	84.17	83.63
Data augmentation β	52,530	97.70	81.84	**84.32**	83.06
Data augmentation γ	9846	97.50	81.02	81.73	81.37
Data augmentation θ	17,927	97.54	81.13	82.30	81.71

### Application in natural product classification

GTC has been demonstrated to be effective in extracting molecular features. We also apply it to other prediction tasks to assess its generalization ability. Here, GTC is employed to classify natural products, utilizing Dataset D in the fivefold cross-validation experiments. Since the data size is sufficient, transfer learning is not employed. The entire model is trained from scratch.

The metrics in Table 4 demonstrate that GTC could effectively classify the natural products into their related pathways in NPClassifier. The highest accuracy score was 100.00% for alkaloids and the lowest score was 98.42% for shikimates and phenylpropanoids. The results imply that GTC could extract molecular features for various applications, such as the creation of novel molecular descriptors and molecular property prediction.

**Table 4:** Performance of GTC in natural product classification

Category	Accuracy (%)	Precision (%)	Recall (%)	F1_score (%)
Alkaloids	**100.00 ± 0.00**	**100.00 ± 0.00**	**100.00 ± 0.00**	**100.00 ± 0.00**
Amino acids and Peptides	98.91 ± 0.22	98.98 ± 0.32	99.80 ± 0.17	99.39 ± 0.12
Carbohydrates	99.06 ± 0.17	99.24 ± 0.25	99.64 ± 0.26	99.44 ± 0.11
Fatty acids	98.85 ± 0.28	98.97 ± 0.42	99.63 ± 0.16	99.30 ± 0.18
Polyketides	98.85 ± 0.28	98.34 ± 0.62	99.48 ± 0.21	98.90 ± 0.26
Shikimates and Phenylpropanoids	98.42 ± 0.55	97.98 ± 1.33	98.38 ± 1.01	98.17 ± 0.62
Terpenoids	99.09 ± 0.16	98.41 ± 0.95	98.55 ± 1.13	98.47 ± 0.29

The values represent the mean ± standard deviation on fivefold cross-validation. The best value on the metric is highlighted in bold, while the lowest value on the metric is underlined

### The EXE program of plant secondary metabolic pathway prediction.

To make the model more accessible, an executable (EXE) program with a graphical user interface (GUI) has been developed as shown in Additional file 1: Fig. S4. Users can input the SMILES string of a query compound, and the program executes the prediction task, displaying the results in the interface and saving it to a text file. The detailed process is demonstrated in Additional file 1: Fig. S5.

## Conclusions

In this study, we propose GTC, a deep transfer learning model that employs a pre-trained hybrid deep learning architecture and transfer learning for the prediction of plant secondary metabolic pathways. GTC mainly consists of two blocks, which leverage a graph transformer-based graph neural network and a convolution neural network to comprehensively learn molecular features. By combining these two representations, GTC provides a more comprehensive representation of molecules, leading to superior performance compared to state-of-the-art methods in predicting metabolic pathways. GTC is pre-trained on the KEGG dataset and then fine-tuned to complete the accurate prediction of plant secondary metabolic pathways. The modular architecture of GTC allows for easy adaptation to other molecular prediction tasks beyond metabolic pathway classification, such as classifying natural products, by modifying the output layer accordingly. This study highlights the potential of deep learning in pathway prediction and offers an accessible tool for predicting plant secondary metabolic pathways.

### Supplementary Information

**Additional file1**. Evaluation metrics, implementation details and supplementary figures.

**Additional file2**. Supplementary tables.

## Data Availability

The EXE program of Plant Secondary Metabolic Pathway Prediction.exe is freely available to academics at https://github.com/ucasaccn/PSMPP.

## Abbreviations

CNN: Convolution neural network
EXE: Executable
FC: Fully connected
GAT: Graph attentional network
GCN: Graph convolutional network
GNN: Graph neural network
GTC: Graph transformer and CNN
GUI: Graphical user interface
KEGG: Kyoto Encyclopedia of Genes and Genomes
PWN: Plant metabolic network
RF: Random forest
SMILES: Simplified molecular-input line-entry system
SVM: Support vector machine
