# Supplementary material for: Prediction of plant secondary metabolic pathways using deep transfer learning
Source: BMC Bioinformatics. 2023 Sep 19;24:348. doi: 10.1186/s12859-023-05485-9 (PMC10507959; doi:10.1186/s12859-023-05485-9)
Supplement: Supplementary file 1 — Additional file1. Evaluation metrics, implementation details and supplementary figures. [file 12859_2023_5485_MOESM1_ESM.pdf]

Supplementary information

Prediction of plant secondary metabolic pathways using deep transfer learning

Han Bao[1,2,3], Jinhui Zhao[1,2,3], Xinjie Zhao[1,2,3], Chunxia Zhao[1,2,3], Xin Lu[1,2,3*], Guowang Xu[1,2,3*]

[1]CAS Key Laboratory of Separation Science for Analytical Chemistry, Dalian Institute of Chemical Physics, Chinese Academy of Sciences, Dalian 116023, China.

[2]University of Chinese Academy of Sciences, Beijing 100049, China.

[3]Liaoning Province Key Laboratory of Metabolomics, Dalian 116023, P.R. China.

* Corresponding authors:

Prof. Dr. Xin Lu, E-mail: luxin001@dicp.ac.cn. orcid.org/0000-0001-5569-1740

Prof. Dr. Guowang Xu, E-mail: xugw@dicp.ac.cn. orcid.org/0000-0003-4298-3554

Evaluation metrics

After completing the model construction, the model performance is evaluated. There are four outcomes for each prediction made by the deep learning model: true positive (TP), true negative (TN), false positive (FP), and false negative (FN). In this study, four widely used metrics are adopted including accuracy, precision, recall, and F1_score, which have been commonly used in previous studies [1, 2]. These metrics are calculated according to the following formulas:

$$Accuracy = \frac{TP + TN}{TP + FN + FP + TN},$$

$$Precision = \frac{TP}{TP + FP},$$

$$Recall = \frac{TP}{TP + FN},$$

$$F1\_score = \frac{2 \times Precision \times Recall}{Precision + Recall}.$$

Accuracy reflects the proportion of correctly predicted samples to the total samples. Sometimes a high accuracy does not mean the model achieves good performance because many true negatives can also make accuracy high. Therefore, precision and recall are required. According to the formulas above, precision and recall focus on true positives regardless of true negatives. precision can reflect the proportion of false positives, while recall can reflect the proportion of false negatives. As the harmonic average of precision and recall, F1_score balances the two metrics and is more resistant to outliers than a simple mean. In this sense, F1-score is a balanced metric for assessing the correctness of models in a variety of domains.

Implementation details

We use Pytorch [3] framework to build a deep learning model. The training process is accelerated on a NVIDIA GeForce RTX 4090 GPU. To ensure better generalization, the default batch size is set as ‘5000’ and ‘1500’ for training and testing, respectively, which allows all the samples to be trained at one time. In the first Graph Transformer layer, an eight-head attention mechanism is adopted, while in the second layer, one head is used. For the two one-dimensional convolutional layers and the two one-dimensional pooling layers, the kernel and the stride are set to ‘3’ and ‘2’, respectively. To better update the weights of the network, the Adam optimizer is adopted.

During the process of model construction, the default learning rate parameter starts at ‘0.0003’, and an early stopping strategy is used to save the best model in 300 epochs. Due to the unbalanced multi-labeled data, the Asymmetric Loss function [4] was applied to adjust the contribution of positive and negative samples to the loss value. This is implemented by assigning different values to the two parameters of gamma_neg and gamma_pos. The value of ‘2’ for gamma_neg and ‘1.5’ for gamma_pos was set because the highest Accuracy and F1_score can be yielded (**Fig. S6**).

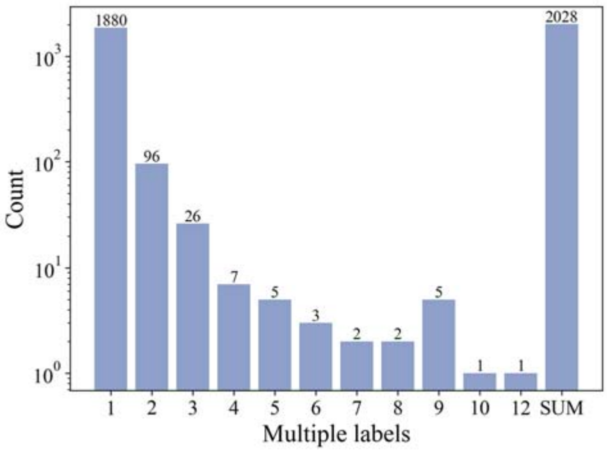

Fig. S1. Distributions of 2028 compounds across multiple labels of plant secondary metabolic pathway classes.

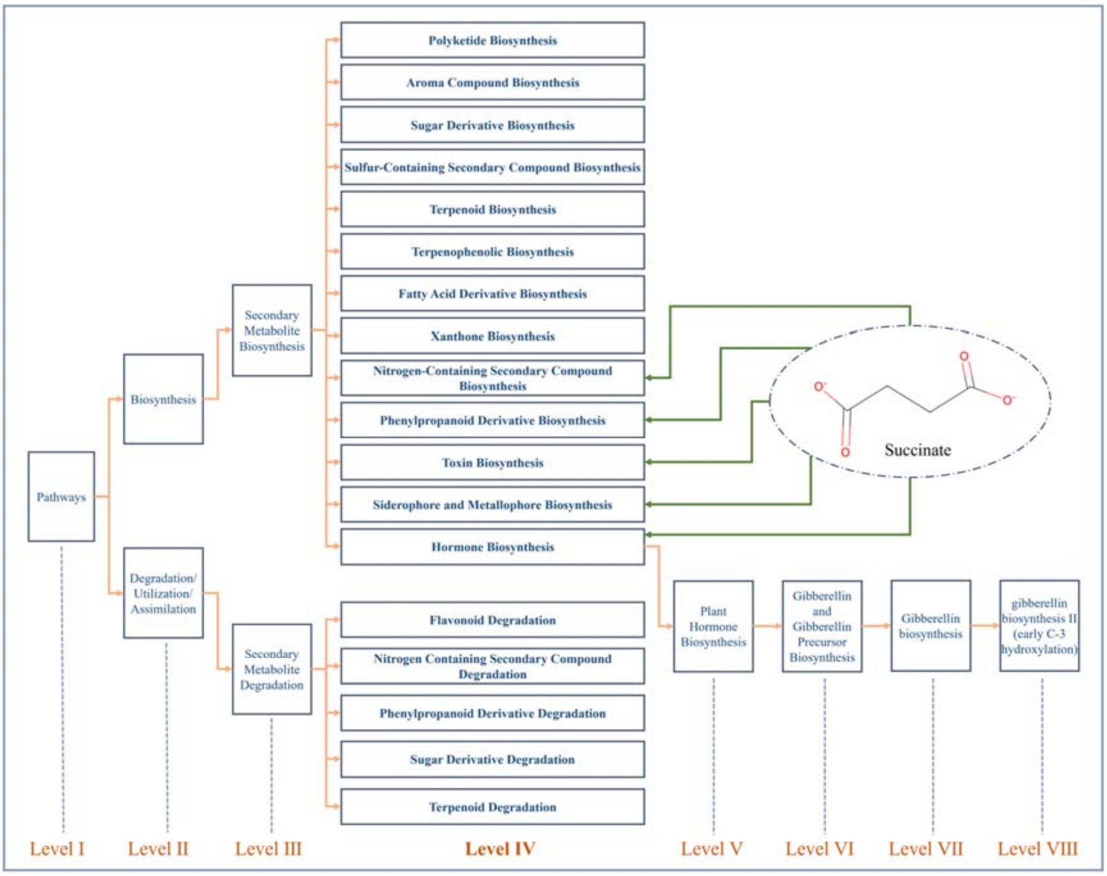

Fig. S2. The plant secondary pathway ontology in PlantCyc using succinate as an example, which arranges plant secondary metabolic pathways hierarchically with a maximum of eight levels. This study specifically focuses on Level IV of the ontology.

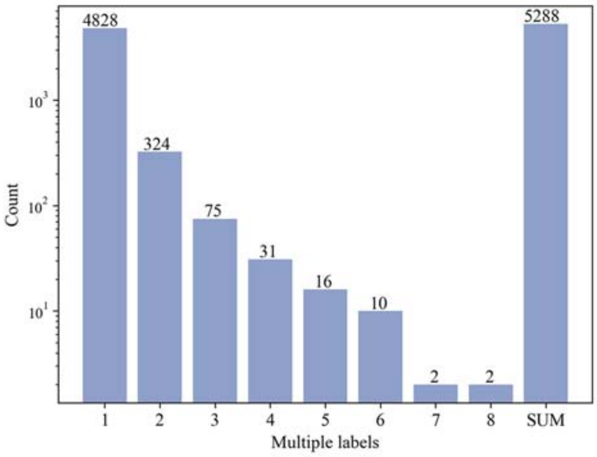

Fig. S3. Distributions of 5288 compounds across multiple labels of KEGG metabolic pathway classes.

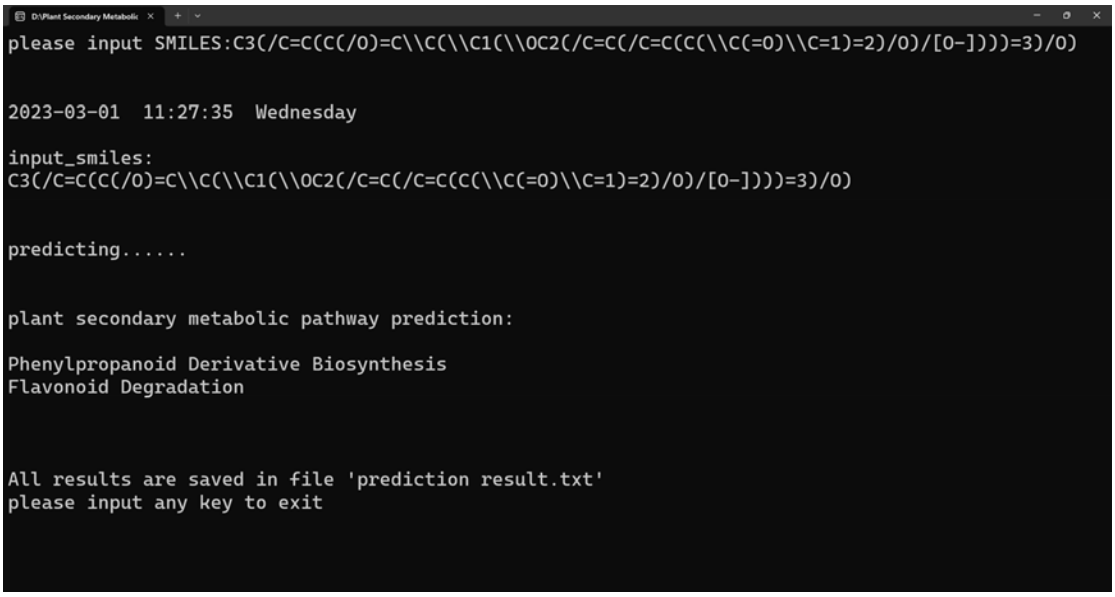

Fig. S4. Interface screenshot of Plant Secondary Metabolic Pathway Prediction.exe.

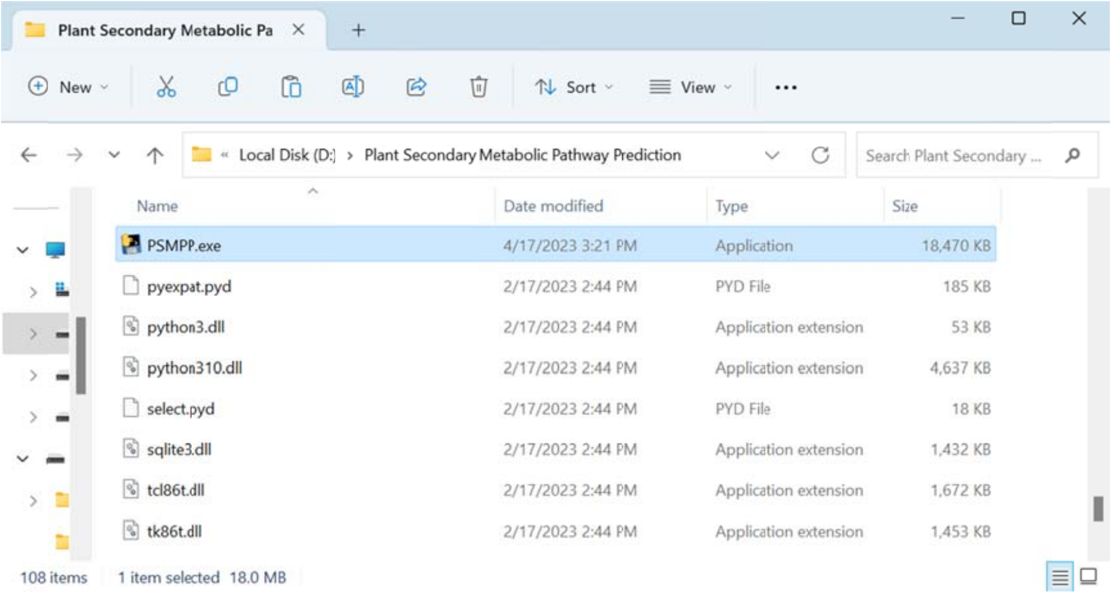

Fig. S5. Software operation flow.gif.

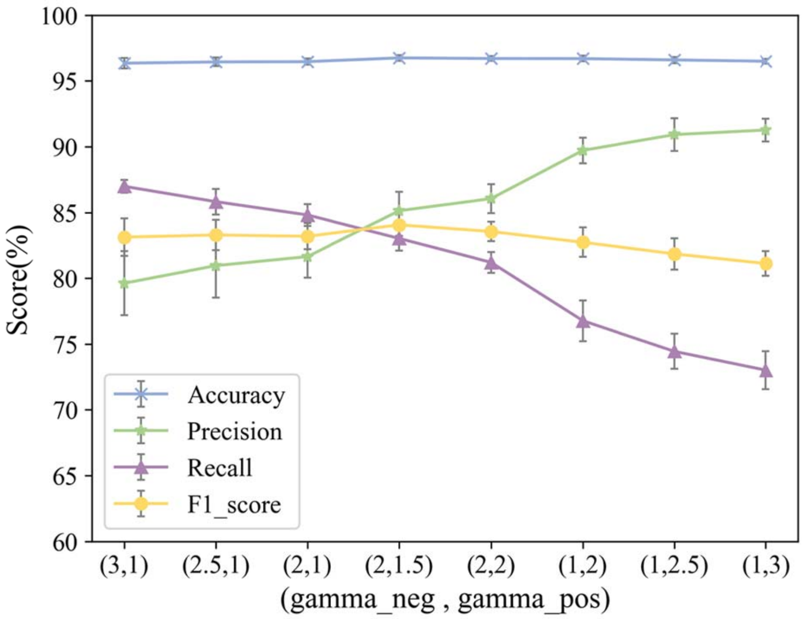

Fig. S6. Performance analysis on the parameter of “gamma_neg” and “gamma_pos” in Asymmetric Loss function.

References

1. Yang Z, Liu J, Wang Z, Wang Y, Feng J. Multi-Class Metabolic Pathway Prediction by Graph Attention-Based Deep Learning Method. In: 2020 IEEE International Conference on Bioinformatics and Biomedicine (BIBM). 2020. p. 126–31.
2. Du B-X, Zhao P-C, Zhu B, Yiu S-M, Nyamabo AK, Yu H, et al. MLGL-MP: a Multi-Label Graph Learning framework enhanced by pathway interdependence for Metabolic Pathway prediction. Bioinformatics. 2022;38 Supplement_1:i325–32.
3. Paszke A, Gross S, Massa F, Lerer A, Bradbury J, Chanan G, et al. PyTorch: An Imperative Style, High-Performance Deep Learning Library. In: Advances in Neural Information Processing Systems. Curran Associates, Inc. 2019. p. 8024–8035.
4. Ridnik T, Ben-Baruch E, Zamir N, Noy A, Friedman I, Protter M, et al. Asymmetric Loss for Multi-Label Classification. In: Proceedings of the IEEE/CVF International Conference on Computer Vision. 2021. p. 82–91.
